# Lomerizine therapy for the treatment of benign paroxysmal vertigo of childhood transitioning into atypical basilar migraine: A case report

**DOI:** 10.3892/etm.2013.1035

**Published:** 2013-04-02

**Authors:** YUTA INOUE, TAKAO YABE

**Affiliations:** Department of Otolaryngology, Tokyo Metropolitan Hiroo Hospital, Tokyo 150-0013, Japan

**Keywords:** benign paroxysmal vertigo of childhood, basilar migraine, lomerizine

## Abstract

We report a rare case of benign paroxysmal vertigo (BPV) of childhood transitioning into basilar migraine (BM) that was effectively treated with lomerizine. A 6-year-old male visited our hospital complaining of repeated attacks of vertigo for 3 months. The patient’s vertigo attacks lasted for several hours and were accompanied by nausea, vomiting, intense fear and loss of consciousness. No nystagmus was observed during the vertigo attacks. Blood tests and imaging examinations revealed no abnormal findings. The results of electronystagmography and the caloric test were unremarkable. Pure-tone audiometry revealed profound right-side sensorineural hearing loss. Among the differential diagnoses, delayed endolymphatic hydrops, epilepsy and BM were considered. Delayed endolymphatic hydrops was considered unlikely since no nystagmus occurred during the vertigo attacks and there was no change in hearing; electroencephalography revealed no epileptic seizure waves. The attacks of vertigo were well-controlled with lomerizine. The patient was diagnosed with BM since the use of lomerizine, an agent for the treatment for migraine, was effective. Since it was reported that BPV is closely related to migraine and the onset of the vertigo attacks was accompanied by a loss of consciousness, we concluded that this patient had BM transitioning from BPV.

## Introduction

Vertigo in childhood is not easy to diagnose since this rare disorder has a number of causes, ranging from traumatic, infective and malignant disorders of the central nervous system to a wide variety of otological disorders, including migraine, benign paroxysmal vertigo (BPV) and psychosomatic disorders. BPV of childhood is defined as recurrent attacks of severe vertigo resolving spontaneously after minutes to hours and is referred to as a periodic syndrome of childhood, migraine equivalent, or migraine precursor (1,2). Reports of BPV are more common in the West than in the East (3). Basilar migraine (BM) is a type of migraine commencing with visual disturbance, vertigo, ataxia, slurred speech, tinnitus, sensory disturbance and occipital headache (1,4). Patients with BM aged <10 years are quite rare. In the current study, we treated a 6-year-old male with BPV transitioning into atypical BM.

## Case report

A 6-year-old male visited our hospital after experiencing repeated attacks of vertigo for 3 months. The patient experienced vertigo forcing the adoption of a crouching position to maintain balance once every few months for several years. The attacks of vertigo lasted for several hours and were accompanied by nausea, vomiting, intense fear and loss of consciousness continuing for ∼30 sec to 1 min immediately after the onset of vertigo. A diagnosis of delayed endolymphatic hydrops had been made by a previous physician and the patient was taking isosorbide, although the symptoms had not improved. The patient’s mother had a history of migraine for which triptan nasal spray was effective.

**Figure 1:**
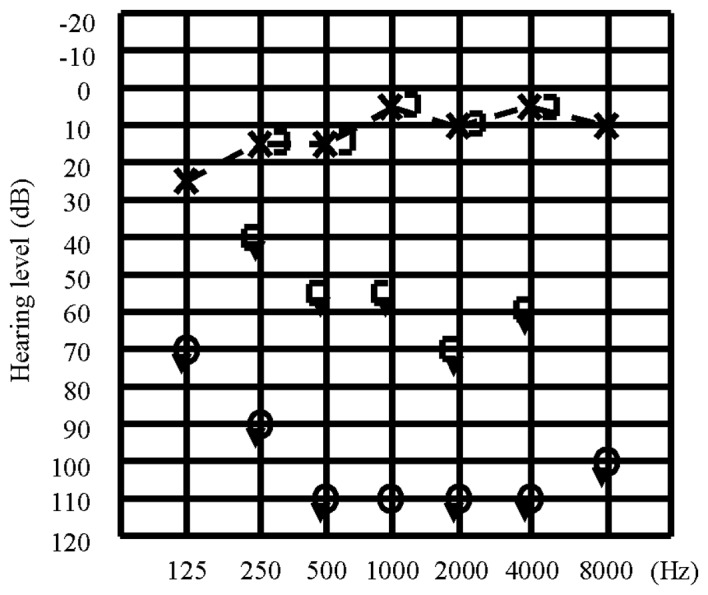
Pure tone audiogram revealing that the left-side was normal and the right-side had profound deafness.

No nystagmus was observed during the vertigo attacks. Blood tests and cervical spine X-rays, as well as computed tomography and magnetic resonance imaging scans of the brain revealed no abnormal findings. The results of electro-nystagmography and the caloric test were unremarkable in the ears. Pure-tone audiometry revealed profound right-side sensorineural hearing loss (SNHL; Fig. 1). Auditory brain stem responses to 105 dB nHL clicks were absent in the right ear.

Among the differential diagnoses, delayed endolymphatic hydrops, epilepsy and BM were considered. Delayed endolymphatic hydrops was considered unlikely since the caloric test results were unremarkable, no nystagmus occurred during the vertigo attacks, the loss of consciousness during vertigo attacks was an atypical symptom, there was no change in hearing and the treatment for delayed endolymphatic hydrops demonstrated no effect. In addition, electroencephalography revealed no epileptic seizure waves. The patient became aware of the unilateral SNHL before the patient’s family noticed. After the family reported right-side SNHL, subclinical mumps infection was diagnosed based on elevated levels of mumps virus IgG antibodies, although there were no clear clinical symptoms of mumps. Finally, the patient was considered atypical BM due to the attacks of vertigo. The vertigo forcing the adoption of a crouching position once every few months was considered to be a manifestation of BPV due to the unremarkable electronystagmographic and electroencephalographic results. Since the attacks of vertigo were accompanied by a loss of consciousness, we concluded that this patient had atypical BM transitioning from BPV.

**Figure 2:**
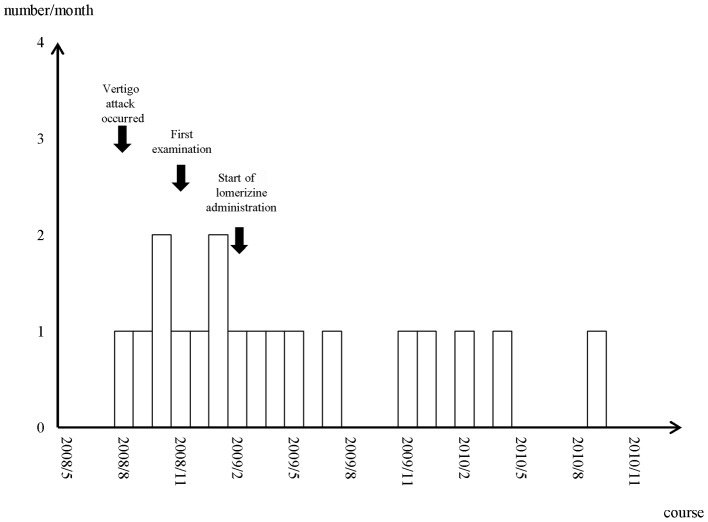
Changes in number of attacks of vertigo. The number of attacks decreased following the initiation of lomerizine.

Based on our diagnosis, lomerizine (5 mg) administration was initiated to prevent the attacks and cyproheptadine (2 mg) was added during attacks. The number and severity of vertigo attacks was reduced following the initiation of therapy (Fig. 2). The patient’s attacks of vertigo are currently well-controlled with lomerizine. Informed consent was obtained from the patient’s family.

## Discussion

According to the International Classification of Headache Disorders second edition (ICHD-II), BPV is defined as recurrent (>5) attacks of severe vertigo resolving spontaneously after minutes to hours (1). BPV is not accompanied by hearing loss and thus we regarded the patient’s unilateral SNHL as congenital deafness or mumps-related deafness.

BPV was defined by Basser in 1964 (2). BPV begins early, at 1–4 years old and is equally distributed across genders. Vertigo is associated with nystagmus, ataxia and other accompanying signs, including phonophobia, photophobia and visual disturbances (5). Russell and Abu-Arafeh reported that the prevalence of BPV is ∼2.1% (6). BPV has a good prognosis and usually disappears with age, although it is occasionally associated with migraine later in life. In the present study, the patient’s mother had migraine; BPV in patients with a family history of migraine may have similar clinical features and trigger factors (6). It has been reported that BPV is closely related to migraine; however, only 4 cases of BM transitioning from BPV have been reported. Golden and French identified that 1 in 8 patients with BM have an original diagnosis of BPV following the first examination (7). Dieterich and Brandt reported that three children with BM in their study could have been diagnosed with BPV following the first attacks (8). Therefore, we conclude that the patient in the current study had BM transitioning from BPV when the vertigo attacks were accompanied by a loss of consciousness.

In the literature, BPV transitioning to BM was observed in 1 of 8 patients in one study (7) and in 3 of 90 patients in another study (8). Therefore, the frequency of transition was 12.5 and ∼3.3%, respectively. Dieterich and Brandt identified a family history of migraine for 71 of 90 patients and of those 71 patients it was the mothers of 29 patients who had migraines (8). Golden and French determined a family history of migraines in 7 of the 8 pediatric patients and 5 of the patient’s mothers had migraines (7). In the present study, the patient’s mother also had migraines, therefore the most important background factor in BPV transitioning to BM appears to be a family history of migraines, particularly in the mother. The trigger of the vertigo attacks remains uncertain; however, an increase in light stimulation may have affected the change in symptoms.

Chang and Young reported that caloric/vestibular evoked myogenic potential (VEMP) test results indicated that upper and lower brain stem lesions occurred in pediatric patients with BPV (9). Dieterich and Brandt indicated that the brain stem was involved as the originating site of vertigo (8). The patient in the present study demonstrated unremarkable responses in caloric testing. Chang and Young also reported that the combined caloric and VEMP test results show abnormalities in 70% of childhood BPV patients (9). Liao and Young identified that 75% of BM patients present abnormalities when the results of caloric and VEMP testing were combined (4). These results did not differ significantly, indicating the similarity of the two disorders. In addition, these results demonstrate that the VEMP test may serve as a diagnostic tool in evaluating vertigo in childhood.

Although BPV attacks are generally so brief that the subsequent administration of drugs is unnecessary, one report commented that analgesics and antiserotonergic drugs may be useful for preventing or treating attacks of headache (5). To the best of our knowledge, this is the first report of lomerizine treatment in a pediatric patient with BPV. Our results suggest that lomerizine administration is one treatment option for particularly severe cases of BPV.

BM was defined by Bickerstaff in 1961 (10). The symptoms include visual disturbances, vertigo, ataxia, slurred speech, tinnitus, sensory disturbances and occipital headache (4). The mechanism of premonitory migraine symptoms was attributed to dysfunction of the brainstem or the occipital cortex. The first BM attack was reported to occur between the ages of 10–72 (mean, 38) years in females and 7–72 (mean, 42) years in males (8). In the literature, few cases of BM developed in patients aged <10 years. Thus, our 6-year-old patient with BM is a rare example.

According to the ICHD-II, headache attack is essential for the diagnosis of BM (1). In the present study, the patient had a stiff neck and shoulders; however, an evident headache did not appear as part of the disorder during the clinical course. Alternatively, the occurrence of headache may not necessarily be required for a diagnosis of BM. Dieterich and Brandt reported that vertigo was not associated with headache in 17 of 90 patients (8). Moreover, the authors suggested monosymptomatic episodic vertigo without accompanying headache and the disappearance of vertigo after receiving treatment for migraine substantiated the diagnosis of migraine. Additionally, Brantberg *et al* identified that 21 of 40 patients with migraine-associated vertigo (MAV) experienced headache with attacks of vertigo (11).

Ergotamine and sumatriptan are contraindicated for the medical treatment of BM since they contract the cerebrovascular system, resulting in the occurrence of aura and other symptoms. Lomerizine, a calcium channel blocker that markedly improves the vertebrobasilar circulation, prevents vertigo attacks. Iwasaki *et al* reported that 27 of 33 adult patients with MAV responded to lomerizine (12). The patient in the present study received lomerizine and the attacks of vertigo are now well-controlled. We administered half an adult dose since the patient was 6-years-old and weighed 24 kg. The frequency of attacks may be reduced by preventive medication with β-receptor blockers, serotonin antagonists, antidepressants and anticonvulsants (8,13).

The patient in the current study appeared to experience attacks of vertigo more often after meals, late at night and early in the morning. Future studies should investigate whether thermal stimulation or brain hypoperfusion affect the onset of vertigo. Lifestyle guidance, including advising patients not to overeat and avoid becoming too cold at bedtime and upon awakening, may be effective in preventing attacks.

In conclusion, we experienced a rare case of a 6-year-old male with atypical BM. Children who present with attacks of vertigo and have a family history of migraine, particularly in their mothers, require long-term observation.
